# Evidence of health system resilience in primary health care for preventing under-five mortality in Rwanda and Bangladesh: Lessons from an implementation study during the Millennium Development Goal period and the early period of COVID-19

**DOI:** 10.7189/jogh.14.05023

**Published:** 2024-07-05

**Authors:** Amelia VanderZanden, Alemayehu Amberbir, Felix Sayinzoga, Fauzia Akhter Huda, Jovial Thomas Ntawukuriryayo, Kedest Mathewos, Agnes Binagwaho, Lisa R Hirschhorn

**Affiliations:** 1 University of Global Health Equity, Kigali, Rwanda; 2 Maternal, Child, and Community Health Division, Rwanda Biomedical Center, Kigali, Rwanda; 3 International Centre for Diarrhoeal Disease Research, Bangladesh (icddr,b); 4 Feinberg School of Medicine, Northwestern University, Chicago, Illinois, USA

## Abstract

**Background:**

The coronavirus disease 2019 (COVID-19) pandemic led to disruptions of health service delivery in many countries; some were more resilient in either limiting or rapidly responding to the disruption than others. We used mixed methods implementation research to understand factors and strategies associated with resiliency in Rwanda and Bangladesh, focussing on how evidence-based interventions targeting amenable under-five mortality that had been used during the Millennium Development Goal (MDG) period (2000–15) were maintained during the early period of COVID-19.

**Methods:**

We triangulated data from three sources – a desk review of available documents, existing quantitative data on evidence-based intervention coverage, and key informant interviews – to perform a comparative analysis using multiple case studies methodology, comparing contextual factors (barriers or facilitators), implementation strategies (existing from 2000–15, new, or adapted), and implementation outcomes across the two countries. We also analysed which health system resiliency capabilities were present in the two countries.

**Results:**

Both countries experienced many of the same facilitators for resiliency of evidence-based interventions for children under five, as well as new, pandemic-specific barriers during the early COVID-19 period (March to December 2020) that required targeted implementation strategies in response. Common facilitators included leadership and governance and a culture of accountability, while common barriers included movement restrictions, workload, and staff shortages. We saw a continuity of implementation strategies that had been associated with success in care delivery during the MDG period, including data use for monitoring and decision-making, as well as building on community health worker programmes for community-based health care delivery. New or adapted strategies used in responding to new barriers included the expanded use of digital platforms. We found implementation outcomes and strong resilience capabilities, including awareness and adaptiveness, which were related to pre-existing facilitators and implementation strategies (continued and new).

**Conclusions:**

The strategies and contextual factors Rwanda and Bangladesh leveraged to build ‘everyday resilience’ before COVID-19, i.e. during the MDG period, likely supported the maintained delivery of the evidence-based interventions targeting under-five mortality during the early stages of the pandemic. Expanding our understanding of pre-existing factors and strategies that contributed to resilience before and during the pandemic is important to support other countries’ efforts to incorporate ‘everyday resilience’ into their health systems.

The coronavirus disease 2019 (COVID-19) pandemic disrupted health service delivery in many countries [1,2], as did the response measures implemented worldwide. The pandemic thus highlighted the importance of health system resilience, whereby countries lacking such health systems have had to deal with persistent challenges on both the demand and supply sides of health service delivery. This has resulted in a rollback in health outcomes and stalled these countries’ progress in under-five mortality outcomes [3]. However, some countries were more resilient in either limiting or rapidly responding to the disruption. Yet resiliency not only reflects a health system’s response to shocks such as COVID-19, but also the maintenance and improvement of the quantity and quality of care delivered before and during these times. While some countries and systems have rebounded well and continued to improve, others have recovered but returned to the poor health service delivery that had existed prior to the COVID-19 pandemic [4].

Despite the realisation that health systems need to function in changing and sometimes unexpected, contexts, health systems and policy research did not emphasise health system resilience until the 2013 Ebola virus outbreak in West Africa, which revealed the inability of health systems to absorb this shock and continue delivering essential health services [5,6]. An important framework by Kruk and colleagues defines health system resilience as ‘the capacity of health actors, institutions, and populations to prepare for and effectively respond to crises; maintain core functions when a crisis hits; and, informed by lessons learnt during the crisis, reorganise if conditions require it’ [7]. Resilience, therefore, is not merely a description of short-term preparedness, but refers to the long-term investments in health systems to provide equitable, quality care both during ordinary times and during crises. It is investments in the building blocks of health systems outside of a crisis that facilitate preparedness – a concept that Barasa and colleagues refer to as ‘everyday resilience’ [8]. While the importance of health system resilience is now understood, the strategies and contextual factors that contribute to resilience capabilities have been less studied, especially prior to COVID-19 [9].

In 2021, the University of Global Health Equity continued an existing collaboration with two countries that experienced success during the Millennium Development Goal (MDG) period (2000–15) [10] in reducing under-five mortality – Rwanda and Bangladesh. From 2017 to 2020, we used implementation research to examine the contextual factors (barriers and facilitators), implementation strategies for evidence-based interventions (EBIs) known to reduce under-five mortality, and outcomes associated with these countries’ successes in lowering it [11–14]. In 2021, we again used an implementation research approach to understand how Rwanda and Bangladesh maintained these key health system-delivered child health interventions during the early period of the COVID-19 pandemic [14–16].

The nature, timeline, and similarity in methods and approaches of the Rwanda and Bangladesh studies in the context of these two time periods (2000–15 and the early period of COVID-19) presents a unique opportunity to identify the mechanisms contributing to health system resilience. Here we focus on understanding similarities and differences in how the two countries leveraged the contextual factors and implementation strategies used to build ‘everyday resilience’ between 2000 and 2015. We also explore what was identified as critical to maintaining the delivery of EBIs during the early period of COVID-19. Our goal is to help contribute to the needed knowledge base on how effective strategies are chosen and applied for resilience.

## METHODS

### Study design and approach

To understand country responses to potential interruptions in service delivery, we conducted a mixed-methods implementation research between February 2021 and March 2022 to explore factors and strategies associated with resiliency in Rwanda and Bangladesh, focussing on how EBIs targeting amenable under-five mortality were maintained during the early period of COVID-19 [11,12,14–16]. We define the period from March 2020 (when Bangladesh and Rwanda reported their first cases of COVID-19) to December 2020 (the end of our study period) as the early COVID-19 pandemic period. The two country case studies highlighted the contextual factors that facilitated or hindered the response to COVID-19-related threats to EBI uptake and delivery; the strategies used to prevent, mitigate, or respond to drops in access to or provision of EBIs; and how decisions were made to continue existing strategies [15,16].

This COVID-19 period study followed an earlier ‘Exemplars in Under-5 Mortality’ project conducted between 2017 and 2020. The University of Global Health Equity, in collaboration with the Exemplars in Global Health programme and in-country research partners, identified and examined how six ‘Exemplar’ countries – Bangladesh, Ethiopia, Nepal, Peru, Rwanda, and Senegal – outperformed their regional and economic peers in reducing amenable under-five mortality between 2000 and 2015, which we refer to as ‘the MDG period’ [11–14,17–19]. Using the same hybrid implementation research framework and a mixed-methods approach, the six case studies identified key facilitating or obstructive contextual factors (i.e. ‘facilitators’ or ‘barriers’), as well as implementation strategies that affected the achievement of key implementation outcomes (including quality and coverage) and contributed to significant reductions in amenable under-five mortality [11–14,17–19].

### Data collection and data sources

**Figure 1:**
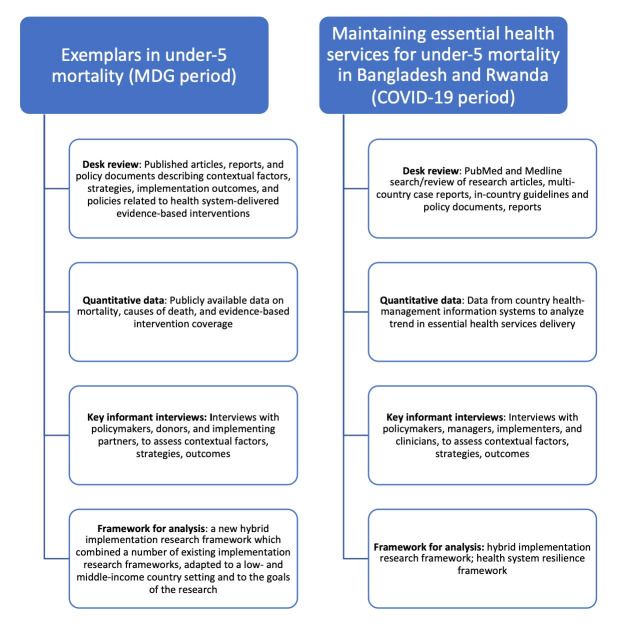
Summary of study methodologies.

We collected data from three sources for each country study during the two periods: a desk review of available documents; quantitative data on under-five mortality, causes of death, and EBI coverage; and key informant (KI) interviews (**Figure 1**). We extracted desk review data from peer reviewed publications retrieved through PubMed and Medline searches, as well as from Ministry of Health policy documents describing strategies, contextual factors, implementation outcomes, and policies related to these health system-delivered EBIs. We analysed existing data from the health management information system and other data sources identified in both Bangladesh and Rwanda. For the COVID-19 period, we explored existing, routinely collected data retrieved from this system, disaggregated by geographic location and granular time unit over the three years before (January 2017 to February 2020) and during the early COVID-19 period (March to December 2020). Across the two study periods, we conducted 68 KI interviews (31 in Bangladesh (18 in the MDG period and 13 in the COVID-19 period) and 37 in Rwanda; (16 and 21 in the respective periods)) with policymakers, managers, implementers, and clinicians using semi-structured interview guides we developed for the first study. We then adapted these guides based on emerging lessons from COVID-19 response and strategies which were associated with preventing, mitigating, or responding to drops in EBI access, uptake, and coverage, as well as Kruk and colleague’s resilient health system framework [7,14].

### Quantitative analysis

We explored quantitative data using descriptive analysis to assess changes in EBIs between the two study periods and used plots to see coverage of selected EBIs in Bangladesh and Rwanda.

### Qualitative analysis

We analysed the KI interview data using the Dedoose software package, version 9.0.17 (SocioCultural Research Consultants, Manhattan, New York, USA) in which we conducted iterative coding to describe relative frequency of categories.

### Analysis and synthesis

To inform the original study design and analysis, we developed a new hybrid implementation research framework which combined a number of existing implementation research frameworks, adapted to a low- and middle-income country setting and to the goals of the research [14]. We triangulated between the desk review, health management information system data, and KI interviews to do a quantitative-qualitative explanatory mixed methods synthesis of contextual factors, implementation strategies and outcomes, and resilience capabilities [20]. We performed a comparative analysis using multiple case studies implementation research methodology, whereby the cases are united by common research questions, facilitating a greater understanding of the set of cases from both commonality and uniqueness [21–23]. We compared contextual factors (barriers or facilitators – a factor, such as geography, could be a barrier in one place (e.g. a country with a large hard-to-access mountain region), yet a facilitator in another (e.g. a small country with a relatively accessible, mostly urban population)), implementation strategies (existing during the MDG period, new, or adapted), implementation outcomes (acceptability, appropriateness, effectiveness, equity, feasibility, fidelity, and reach or coverage), and resiliency capabilities (aware, integrated, adaptive, self-regulating, and diverse). We developed a matrix of all implementation strategies and all contextual factors identified in Bangladesh and Rwanda during the MDG and COVID-19 periods. We used this matrix assess which strategies implemented in the MDG period helped mitigate the drop in EBIs during the early period of COVID-19. We also looked at how the previous efforts to implement these EBIs during the MDG period supported the work to maintain EBIs during the pandemic and contribute to resilience capabilities in the COVID-19 period.

## RESULTS

### Contextual factors

Facilitating contextual factors we found across both countries and both time periods were a culture of collaboration and coordination; health system structures and strength; leadership and governance and a culture of accountability; national priority for health and primary care; pre-existing culture and capacity for data use; and a strong pre-existing community health system and structure. Common challenging contextual factors were largely new and were common across many countries, stemming from COVID-19 and pandemic response: a fear of COVID-19 by the community and by health care workers; lockdown and movement restriction; and workload and staff shortage. There were also several factors unique to the context of Rwanda and Bangladesh: culture and beliefs, health insurance, and pandemic preparedness. While we identified these three factors as barriers in Bangladesh, we either found them to be facilitators in Rwanda or we did not identify them within Rwanda at all. Below we discuss several examples of important contextual factors and expand them in **Table 1**.

**Table 1:** Key contextual factors identified during the MDG and COVID-19 periods in Bangladesh and Rwanda which were facilitators, barriers, or both, and transferable lessons for health system resilience*

	Bangladesh		Rwanda		
**Contextual factor (existing or new)**	**MDG period**	**COVID-19 period**	**MDG period**	**COVID-19 period**	**Transferable lessons/evidence for health system resilience**
Culture and beliefs (existing)	−	−	+	N/I	In Bangladesh, culture and beliefs were a barrier contributing to lower health outcomes in two of the country’s most underperforming districts. A KI explained that ‘even though many of the people living there are quite affluent, the social taboo and care seeking pattern is not upright there. They suffer much from social taboo especially in Sylhet, the entire Sylhet Division and the hilly areas in Chittagong.’
Culture of collaboration and coordination (existing)	+	+	+	+	Bangladesh leveraged its existing network of collaborators to respond to COVID-19 and maintain EBIs. In Rwanda, the existing structure and culture of collaboration between the government, implementing partners, donors, technical working groups, and professional associations through the national strategic plan prevented duplication of efforts. A KI noted the importance of this collaboration, stating that ‘A key of all these activities is the teamwork of all sectors…Private sector, security organs, local leaders, and health sectors and professionals, we worked together, and this was a big and very important thing, that helped that to be as stable as possible during this period.’
Fear of COVID-19 by the community (new)	N/I	−	N/I	−	Fear of COVID-19 was a significant barrier to health seeking behaviour in both countries. In Bangladesh, for example, ‘because people made the decision based on the social taboo that if the women go for facility delivery, then she will get infected with COVID-19,’ facility-based delivery decreased substantially in the first months of the pandemic. Both countries used community education through health communication as a strategy to overcome this barrier.
Fear of COVID-19 by health care workers (new)	N/I	−	N/I	−	In both countries, the novel virus and lack of PPE contributed to HCWs’ fear. Contributions to overcoming this barrier included training on COVID-19, reassurance from leaders, and realisation that COVID-19 patients were surviving in Rwanda; and growing familiarity with the virus, the availability of protocols and antigen tests, and the enforcement of COVID-19 prevention guidelines in Bangladesh.
Health system ownership (existing)	N/I	+	N/I	+	The combination of Rwanda’s culture of culture of coordination of partners with strong leadership at the central and local levels allowed it to guide donor and implementing partners’ funding and project priorities towards the needs of the country. Despite their fears, HCWs were committed to providing care: according to one KI, ‘no employee ever missed work whether during the day or the night and no one ever missed work due to the fear of COVID.’ In Bangladesh, ownership meant that guidelines – for example, WHO recommendations – were adapted to the local context.
Health systems structure and strength (existing)	+	+/−	+	+	In both countries, the organisational structure of the health systems contributed to the continuation of health service delivery. In Rwanda, this included the *mutuelle de santé* (community-based health insurance), human resources for health to strengthen health care capacity, and a cascaded training/mentorship structure. In Bangladesh, a KI explained that ‘the organisational structure of this department [the DGHS] at the field level is the strongest and most trusted,’ which helped ensure that services continued to be provided as normal. However, in Bangladesh, the staff shortage barrier also impacted the health system structure and strength, as it impacted the quality of health care delivery.
Lockdown/movement restriction (new)	N/I	−	N/I	−	In Rwanda, a KI noted that during the first lockdown ‘gathering was not allowed and so [there were] problem[s] for CHWs’ reporting of daily activities.’ However, as various strategies were implemented to address the challenges to health service delivery created by the lockdown, these challenges subsided. A KI noted that they did not face this same level of challenge during the second lockdown in January 2021 as they had learned, and adapted and adopted new strategies, in response to the first one.
National priority for health and primary care (existing)	+	+	+	+	Rwanda has a decentralised health care system, with each health care level providing more comprehensive care than the facilities at the lower level. Clear lines of communication and referral links between the different levels of the health system were established to ensure timely referral of patients needing more specialised care. This structure has allowed the health sector to increase the reach of the health system into remote corners of the country.
Pandemic preparedness (existing)	N/I	−	N/I	+	Several KIs cited Bangladesh’s lack of preparedness for a pandemic as a challenging factor. One explained that this was due to the health system’s approach: ‘pandemic preparation or public health preparation, whatever you say, there is a lot of lacking. Because the system is still clinical service oriented, it will take us a long time to make it public health oriented.’ Conversely, in Rwanda, preparedness was specifically cited as a facilitating factor: the system built prior to the pandemic, based on experiences with Ebola Virus and H1N1, included plans that could be implemented swiftly when the country responded to COVID-19.
Pre-existing culture and capacity of data use (existing)	+	+/−	+	+	In both countries, a culture and capacity of data availability, quality, and use was an important facilitator, allowing the governments to track coverage of EBIs, and make well-informed decisions. In Rwanda, a centralised data reporting system brings all governmental, non-governmental, and private facilities reports into the HMIS; a Rapid SMS System additionally provides real time data on matters including pregnancy and nutrition. A challenge in Bangladesh was the lack of integration between public and private facility data. A KI explained: ‘We talk a lot about data; unfortunately, all are public data, not a single one is private data. And you will never get a total picture until the total data system is integrated.’
Strong supply chain system (existing)	N/I	+/−	N/I	+	In Bangladesh, some noted that the strength of the supply chain system prevented stockouts; others pointed out that weaknesses in the system were exposed by the pandemic: for example, one KI stated that ‘when it comes to this evidence-based intervention for maternal, newborn and child health, there was no smooth flow of logistics up to the primary health care center level and that kind of weakness actually emerged during the COVID as well.’ In Rwanda, the supply chain was able to adapt to new circumstances, including for example moving to a push rather than pull system for the delivery of medicines from the central level.
System of learning and improvement (existing)	N/I	+	N/I	+	In Rwanda, regular monitoring and evaluation supported the identification of disrupted health services, and informed decision-making at all levels. The country was able to implement strategies to leverage this, for example, one KI explained: ‘in one month, we realised that the decrease in health-seeking behaviour, we designed a strategy for the community to seek treatment [from] CHWs.’ In Bangladesh, the government was kept informed by real-time data analysis, and regular feedback and monitoring meetings tracked progress and facilitated improved strategies.

CHW – community health worker, DGHS – Directorate General of Health Services, EBI – evidence-based intervention, HCW – health care worker, HMIS – health management information system, HR – human resources, KI – key informant, MDG – Millenium Development Goal, MNCH – maternal, newborn, neonatal, and child health, PPE – personal protective equipment

*Plus (+) symbol denotes a facilitator; the minus (−) symbol denotes a barrier; the plus/minus (+/−) denotes both a facilitator and a barrier simultaneously; N/I denotes not being defined/not being identified as having an impact.

#### Leadership and governance and a culture of accountability (common facilitator)

In Bangladesh, leadership made a commitment to maintaining EBIs at all levels, and collaboration between ministries facilitated the response to COVID-19. A KI explained:

There was a commitment from the national level like from the Ministry of Health or DGHS that we should keep running these services even in this pandemic situation. And, as a whole, those who were our stakeholders, also declared their solidarity with us that how we would keep continuing the services in this situation.

In Rwanda, a commitment to serve the vulnerable was critical during COVID-19, as vulnerable populations were most affected. A culture of accountability beyond the health sector included political leadership at district levels, for example *imihigo* contracts (performance contracts that district mayors sign with Rwanda’s president), as one KI explained:

It was our task as policymakers to ensure that if we are preaching about or telling people to ensure that there is no disruption in essential health services, we made sure that there was no stock-out.

The resulting trust increased adherence to public health measures and continued uptake of health services during the pandemic.

#### Strong pre-existing community health system and structure (common facilitator)

In Rwanda, the community health system was responsible for communicating the continuation of health services. Community health workers (CHWs) were mentored and supervised by health center staff and trained to provide services. A KI explained that CHWs:

[…] meet regularly on monthly basis so it is a time for them when they meet at health center to discuss on issues, observe where they were treating or where they can correct themselves if there is some error occurring during their practices.

The existing CHW system in Bangladesh allowed for service delivery at the household level in response to reduced access at the facility level, with health education and support of delivery of health services rapidly expanding at the community level before and during the pandemic. Community health workers were critical to efforts aimed at raising awareness about COVID-19 and pushing for COVID-19 prevention measures among communities. In terms of service delivery, a local service partner (BRAC) started providing family planning, child health service, and immunisation at the household level to respond to the reduction in service utilisation at the facility level.

#### Workload and staff shortage (common barrier)

The health system in both countries refocussed on the COVID-19 response, with health care workers moving from their usual positions to centre on providing COVID-19 treatment. In Rwanda, where workload and staff shortage was a new barrier, health care workers were overwhelmed in the early days of the pandemic with the extra burden of COVID-19. Separate COVID-19 treatment centres were established, and some health care workers had to physically relocate, while the remaining ones had to work overtime and were asked by the Ministry of Health to avoid taking vacations or leave. A KI explained that, besides the increased workload, *‘*the services cannot be the same level as previous[ly] it was.’ Health care workers overwhelmed with additional responsibility ultimately received additional support from the government and partners.

In Bangladesh, pre-existing staff shortages were exacerbated by the pandemic. One KI explained how this affected service quality:

The huge HR shortage [is] still kind of a valid factor… actually hampering the quality service delivery. Whenever we are working at the facility level, this issue is coming up as one of the critical barriers in terms of provision of quality of care, quality of MNCH services.

#### Health insurance (facilitator in Rwanda, barrier in Bangladesh)

In Bangladesh, the lack of a social safety net such as universal health coverage meant that the poor community members were increasingly vulnerable. A KI noted:

I think this is a critical area because we don't have that kind of financial support mechanism in the country to support the poorest of the poor, in particular. Poor are getting poorer during this COVID-19 situation.

This was previously identified as a barrier in the MDG period, when low health insurance coverage and high out-of-pocket expenditure were identified as challenges to reducing under-five mortality.

By contrast, health insurance in Rwanda was identified as a facilitator during the MDG period, with the establishment of community-based health insurance in 2004. In 2020, around 90% of Rwanda’s population had community-based health insurance, which provided free COVID-19 services among other benefits. One KI explained:

We still put emphasis on strengthening the primary healthcare... the services related to COVID-19 were free and now are part of the mutuelle [community-based health insurance].

### Implementation strategies

In both countries, we saw examples of continuity of several implementation strategies for delivering EBIs targeting amenable under-five mortality that had been implemented during the MDG period. These included building on CHW programmes for community-based health care delivery; community engagement and education; data use for monitoring and decision-making; donor and implementing partner coordination and engagement; focus on equity; human resources for health; and training, mentorship, and supervision (**Table 2**). New strategies, either newly adopted during the COVID-19 period or newly identified from the MDG period, included a direct response to COVID-19 and support to maintain EBIs; enacting policies and guidelines to support the maintenance of essential health services; leveraging existing programmes and systems; provision of transport; and use of digital platforms and e-health. We discuss key examples of continued or adapted strategies common across both countries and time periods, and provide more details in **Table 2**.

**Table 2:** Implementation strategies identified in the MDG and COVID-19 periods in Bangladesh and Rwanda, whether they were new, adapted, or continued, and transferable lessons for resilient health systems

	Bangladesh		Rwanda			
**Implementation strategy**	**MDG period**	**COVID-19 period**	**MDG period**	**COVID-19 period**		**Transferable lessons/evidence for health system resilience**	
Community engagement and education	Existing strategy	Continued	Existing strategy	Adapted/continued		Ensure timely and relevant information and communication: KIs emphasised the critical role of providing timely information to patients, health care workers, and communities during this COVID- 19 period. The provision of timely information helps disseminate accurate information related to disease transmission, disease prevention and care, availability and access to EHS, increase public trust and alleviate fear towards the disease. A KI Rwanda explained that ‘communication was a big contribution because everything was transparent, the number of cases was communicated every day, this helped people to know how things are moving, and it gives more trust in the community.’	
						Community engagement and civil society engagement: KIs from Bangladesh emphasised engaging the community and civil society as key strategy in the effective response to COVID-19 as well as mitigating the drop in EBI delivery. One KI said: ‘I think the model that we have followed in our organisation is a better one. We were always with the government, every one of us. We have tried to give all possible support to all our members from the central level, even when a girl fallen ill up to guidelines development.’	
Data use for monitoring and decision-making	Existing strategy	Adapted/continued	Existing strategy	Adapted/continued		Use data for decision-making at all levels of the health system and work to invest in and build an information system which ensures the availability of quality data: In both countries, the strategy of data use for monitoring and decision-making was commonly identified by KIs across both MDG and COVID-19 periods. Actions done before COVID-19 pandemic were important in mitigating the disruption of essential health services during the period COVID-19 pandemic. Before the pandemic, for example, Rwanda had a centralised data electronic and real-time reporting system using its HMIS platform and rapid SMS which facilitated data use to identify challenges and drive decisions related to EBIs and timely communication of COVID-19 response. A KI explained: ‘That's what Rwanda doe[s]...flexible to learn from science also to learn from our culture, then to develop our own strategy, not depending on only evidence done elsewhere, but evidence combining international research evidence mixed with our culture, to develop our home-made solutions.’ In Bangladesh, weekly monitoring meetings organised by the Directorate General of Health Services with partners and collaborators focussed on data review and feedback to areas of low coverage. A KI explained: ‘Our main focus was data analysis to see how we adopt policy and take management decisions for operational monitoring. The purpose of the MNCH operation plan monitoring service was to monitor the operation and support in decision-making.’	
Direct response to COVID-19 and support to maintain EBIs	Not defined/not identified	New	Not defined/not identified	New/adapted		Use the response for broader health system strengthening: Bangladesh leveraged the response to COVID-19 to establish and develop oxygen production capacity that could be used for both COVID-19 and non-COVID-19 patients. This also supported non-COVID-19 cases including management of pneumonia. A KI said: ‘We had a global webinar particularly on this oxygen element because this is a learning for the other countries, how quickly Bangladesh adapted to the situation first, building its oxygen capacity and how quickly it integrated within the MNCH programme. This might be the learning for many other countries.’	
Donor and implementing partner coordination and engagement	Existing strategy	Continued	Existing strategy	Adapted/continued		Ensure coordination of activities across partners and sectors. A strong system of coordination of activities between MOH, donors, and implementing partners including multi-sectoral collaboration supported the implementation of EBIs during the pandemic. The work of donors and implementing partners towards one plan during the period of COVID-19 was well coordinated and managed in Rwanda by the MOH using existing mechanisms of coordination and communication including technical works groups and command posts. One KI said: ‘This response is multi-sectoral. So, we need everyone who should be involved… understanding their role at it, and working towards achieving a common goal. So, it's not something we can leave to the health sector… we definitely need all those that need to be on board, and… all of them working together towards a common goal.’ A KI in Bangladesh provided a direct example of this collaboration, stating that ‘We have been working with DGHS from the beginning and we have been trying to figure out if there is a gap and what compliments can be made and we did it. For example, we worked at the beginning of the COVID-19 sample collection, and then DGHS showed the concern that sample collection in the community outside of the health center is not even possible for them, meaning they were struggling thinking how they could do it. Then they told us “Can you do it?” We agreed, because we work in the community.’	
Enacting policies and guidelines to support EHS maintenance	Not defined/not identified	New/ Adapted	Not defined/not identified	New/adapted/continued		Develop and implement strong guidelines and procedures, with timely communication and with national leadership and ownership: KIs emphasised the role of the development of guidelines and procedures, and effective communication of the guidelines at various levels was key in the response to COVID-19 and to mitigate EBI drops in Bangladesh. This guideline provided a clear strategy on how to continue serving children under five during COVID-19 situation. A KI explained that ‘if I don’t have the guideline, how can I provide the services? You cannot do it haphazardly. So, a clear-cut direction is needed for the service providers.’	
Focus on equity	Existing strategy	Adapted/continued	Existing strategy	New/adapted/continued		Focus on equity: Rwanda focussed on an equity agenda to identify and support the most vulnerable during the period of COVID-19. This was determined through disaggregated data analysis by geography, and districts with low coverage of EBIs were supported with money redirected from programmes to support vulnerable populations and nutritional support. A KI explained that if ‘we promote or want to achieve universal health coverage, people should come first. Basically, see that people are accessing services equitably but also without incurring any financial barriers to get services.’	
Human resources for health strengthening	Existing strategy	Adapted	Existing strategy	Adapted/continued		Respond to human resources for health needs: In both countries, workload and staff shortages were a challenge to providing timely health services delivery. In Bangladesh, the government gradually increased HR deployment, recruiting additional health workers. Rwanda used and adapted existing strategies including recruitment of new staff and volunteers, restructuring staff from health centers to health posts to increase outreach services, particularly for vaccination, and adapting the roles of health care professionals at facilities to make up for those sent to staff COVID-19 treatment centers. Budgets were reallocated to districts that needed more staff. Mentorship and supervision of CHWs stopped in the beginning but were adapted, and continued through online channels to maintain quality of services delivered.	
Leveraging existing programmes and systems	Not defined/not identified	Continued	Not defined/not identified	Adapted/continued		Invest in health systems, inputs, and quality: Most KIs in Rwanda reported the presence of a strong primary health care system and investing in diverse health system inputs and quality at all levels of care (central, district, and community level) were critical to respond or mitigate disruptions of EHS during the COVID-19 pandemic. This included building a strong primary and community health system and investment in infrastructure, HR, and supply chain. KIs emphasised the need to build a strong and resilient health system which would allow a country to respond to any epidemic based on country context as well as in-country capabilities. A KI explained that a country should ‘build a strong health system at all levels, from community health workers up to the level of hospitals. So, if, prior to any pandemic, you build that system to be strong enough to function, it will be able to respond to emergencies that can arise.’	
Provision of transport	Not defined/not identified	Adapted	Not defined/not identified	New/adapted		In both countries, provision of transport to health care workers and in some cases patients was a new strategy to overcome the challenge of movement restrictions. A KI in Bangladesh explained: ‘Since we have doctors or nurse living far and to move amid the lockdown situation, transport facilities were provided.’ In Rwanda, existing strategies, including the provision of vehicles and motorbikes to health centres to support vaccination programmes, provision of ambulances to patients, and provision of vehicles to bring those people trying to go for health care services at different health facilities particularly for mothers who gave birth at the hospital and for patients who require referral services, were adapted to meet the needs of the COVID-19 context.	
Supply chain strengthening	Not defined/not identified	Not identified	Existing strategy	Adapted/continued		Rwanda had a strong supply chain system that helped prevent stock-out of essential supplies and medicines during the period of COVID-19, and ensured continuity of EHS reflecting a strategy that began in the early 2000s. Some key implementation strategies which helped prevent stockout of medical supplies included planning well in advance of shipment of supplies to avoid stockout, redesigning the supply chain system, particularly for vaccines using a push system (such as active distribution of vaccines and supplies to districts using refrigerator truck) and continued use of local manufacturers to supply pharmaceutical products due to international disruption of stock supply.	
Training, mentorship, and supervision	Not defined/not identified	Adapted/continued	Not defined/not identified	New/adapted		Various trainings were provided during the period of COVID-19 in Bangladesh in order to respond to the initial disruption of EBIs, including provider training on the transmission of COVID-19 (for example no vertical transmission; no transmission through breastfeeding) so newborn care should continue; training on community nutrition, on hypertension, the Comprehensive Newborn Care Package, and HR-related trainings. In Rwanda, mentorship and supervision were important strategies utilised with some level of adaptation to COVID-19 preventive measures to ensure continuity and delivery of quality EBIs. For example, while some in-person supervision by district as feasible remained, most supervision activities moved to online platforms.	

ANC – antenatal care, CHW – community health worker, DGHS – Directorate General of Health Services, EBI – evidence-based intervention, EHS – essential health service, HR – human resources, KI – key informant, MDG – Millenium Development Goal, MNCH – maternal, newborn, and child health, MOH – Ministry of Health, PNC – postnatal care, PPE – personal protective equipment

#### Use of digital platforms and e-health

The use of digital platforms was critical during the COVID-19 period in both countries. Rwanda used various strategies related to digital technology to continue the delivery of essential health services. Adapted strategies included establishing a WhatsApp group with data managers, CHWs, and supervisors for data audit, feedback and discussion of monitoring and evaluation activities, and maintaining coordination. Professional members also used WhatsApp groups to discuss activities and exchange new information in contexts where face-to-face meetings and discussions were restricted. The use of meeting platforms such as Webex and Zoom virtual meetings with partners and stakeholders (such as technical working groups and Ministry of Health coordinating meetings) facilitated the maintenance of other existing strategies such as donor coordination.

In Bangladesh, digital health services to promote and protect primary health care, including telehealth, video consultation, reporting (even through data collection from the remote rural areas), monitoring, surveillance, and human resource development (including continued professional development) had been underway well before the COVID-19 pandemic. During the COVID-19 period, additional services were added, such as risk communications, contact tracing and ‘hot spot’ identification, and strengthening of telemedicine. Additional adaptations included moving to virtual meetings and trainings using online communications platforms; the use of telemedicine (including phone consultations); and the use of Viber groups for information sharing and communication.

#### Building on CHW programmes and community-based health care delivery

In Bangladesh, KIs explained how important the presence of a strong health system structure, investments in diverse health system inputs, and quality at all levels of care were to the country’s ability to respond to or mitigate COVID-19-related disruptions of EBIs. For example, one KI explained that the structure of the immunisation system, reaching from the central level to outreach centres nationwide, meant that ‘there has never been a shortage of vaccine supply chains in these centres during the pandemic period even during more crisis periods.’ In Rwanda, the community health system is recognised as an important element in the country’s progress towards improved child health outcomes, including through expanded access to primary health care and community-based health insurance. While emphasising the essential role of community health workers in this system, a KI explained:

We [were able to do] a close monitoring of communities because CHWs were working together daily with all the families. Even today we are still in pandemic, they are the ones who are giving vitamins and deworming.

### Implementation outcomes

In **Table 3**, we describe select contextual factors, with relevant implementation strategies, resilience capabilities, and associated implementation outcomes, including acceptability, coverage and reach, fidelity, and equity, during the early period of the COVID-19 pandemic. Community education was identified as an important strategy to increase the acceptability and effectiveness of EBIs in both countries. Coverage, reach, and fidelity, meanwhile, were aided by training, mentorship, and supervision. In both countries, building public trust was an important aspect of community education and engagement, as well as in the outcome of acceptability. In Bangladesh, for example, a KI described how the strong existing community health system interwove with community engagement to build trust and increase acceptability:

**Table 3:** Selected contextual factors, with relevant implementation strategies, resilience capabilities, and associated implementation outcomes during the early period of the COVID-19 pandemic

Contextual factors	Contextual factor role as facilitator or barrier	Implementation strategies to address or leverage contextual factor	Relevant resilience capability [7]	Associated implementation outcome
Culture of collaboration and coordination (facilitator in both Bangladesh and Rwanda)	As a facilitator: Allows for coordinated, frequent engagement and facilitates efficient implementation. As a barrier: Limits engagement and leveraging resources; non-alignment of donors and partners can impact sustainability of implementation.	Leveraging international and in-country partner and donor support; donor and implementing partner coordination and engagement; data use for monitoring and decision-making	Adaptive, aware, self-regulating, integrated, diverse	Appropriateness, acceptability, coverage, equity, reach, sustainability
Health systems structure and strength (facilitator in Rwanda, both a facilitator and barrier in Bangladesh)	As a facilitator: Facilitates integration into and strengthening of existing systems required for efficient implementation. Allows for important community interactions needed for efficient implementation. Aids efficient community-based delivery. As a barrier: Limits implementation unless strategies focussed on strengthening are employed. Where not sufficiently compensated, trained, supervised, or engaged with community, limits accountability and sustainability of implementation.	Direct response to COVID-19 and support maintain EBIs; enacting policies and guidelines to support EHS maintenance; focus on equity; leveraging existing programmes and systems; supply chain strengthening; training, mentorship, and supervision; use of digital platforms and e-health	Adaptive, aware, self-regulating, integrated, diverse	Cost, coverage, effectiveness, equity, feasibility, fidelity, reach, sustainability
Leadership and governance and a culture of accountability (facilitator in both Bangladesh and Rwanda)	As a facilitator: Allows for timely and coordinated leveraging of the resources, engagement, and strengthening required for efficient implementation of EBI. As a barrier: Limits coordination in engagement, strengthening, and leveraging resources which threaten acceptability, feasibility, and sustainable implementation.	Data use for monitoring and decision-making; direct response to COVID-19 and support maintain EBIs; donor and implementing partner coordination and engagement; enacting policies and guidelines to support EHS maintenance; leveraging existing programmes and systems	Adaptive, aware, self-regulating, integrated, diverse	Appropriateness, effectiveness, feasibility, fidelity, sustainability
Pre-existing culture and capacity of data use (facilitator in Rwanda, both a facilitator and barrier in Bangladesh)	As a facilitator: Supports data use for decision-making required for efficient implementation. As a barrier: Limits data use for decision-making required for timely and efficient implementation.	Data use for monitoring and decision-making; direct response to COVID-19 and support maintain EBIs; focus on equity; training, mentorship, and supervision; use of digital platforms and e-health	Adaptive, aware, self-regulating, integrated, diverse	Coverage, effectiveness, equity, fidelity, reach

EBI – evidence-based intervention, EHS – essential health service

Not in many countries, you will see that people are vaccinated in their homes with a temporary vaccination center (during outreach activities)... it clearly reflects that the people of that area own the activities of EPI [the Expanded Programme on Immunization].

Rwandan KIs also highlighted the role of public trust towards health interventions, emphasising that leadership must work purposively towards building it:

If you have a population that actually trusts you… the leadership and the system that is helping the people to go through you know such moments – then it definitely makes the response something attainable without claiming too many lives.

### Resilience

**Figure 2:**
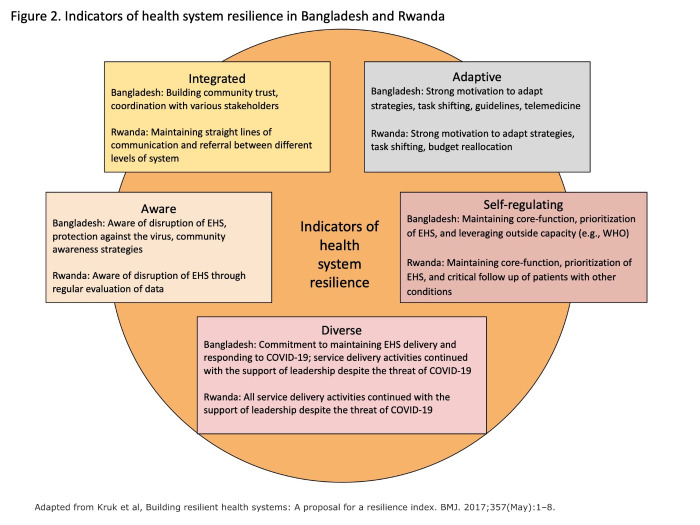
Indicators of health system resilience capabilities in Bangladesh and Rwanda.

**Figure 2** and **Table 3** describe examples of the contributors to the health system resilience framework capabilities identified in the two countries during the COVID-19 period [7]. The pre-existing facilitators identified in the MDG period, such as strong pre-existing community health systems and structures including CHWs, were important contributors to the countries’ abilities to be adaptive, diverse, aware, and integrated. This is also the case for the continued or adapted implementation strategies such as engagement and education of the community and data use for monitoring and decision-making from the earlier period. For example, both countries utilised community engagement and education as a strategy that contributed to an aware system. In Bangladesh, where antenatal care and facility-based delivery fell off significantly in the early months of the pandemic, the engagement and education strategy was important to get people back to using these EBIs, increasing acceptability, coverage, and reach. In Rwanda, where the fall-off in demand for these services was not as profound [15,16], there was a focus on integrated risk communication across existing community interventions and messaging reminders to mothers in the vaccine programmes.

## DISCUSSION

We analysed if and how strategies to implement health system-delivered EBIs targeting amenable under-five mortality that had been present in Bangladesh and Rwanda during the MDG period were maintained during the COVID-19 period. We found that both countries experienced many of the same facilitators at any given time point and over time, and that both experienced new, pandemic-specific barriers during the COVID-19 period. We saw examples of continuity of implementation strategies that had been identified during the MDG period in both countries, as well as new or adapted strategies in response to new barriers. Existing facilitators were likely contributors to the countries’ resilience capabilities such as awareness and adaptiveness; these were leadership and governance, a culture of accountability, and strong pre-existing community health system and structure, but also continued/adapted use of existing strategies such as use of data for decision-making, use of digital platforms and e-health, and building on CHW programmes and community-based health care delivery.

Across both countries and periods, the value of a strong pre-existing community health system and structure, and CHWs in particular, was a repeated theme in our work. Similarly, researchers found that in Malawi, a strong community health strategy with a clear chain of command helped to ensure access to essential health services during COVID-19 [24]. In a review of 28 countries, several reaffirmed the ongoing importance of CHWs in facilitating community awareness and engagement to service delivery functions [25].

Bangladesh and Rwanda were able to leverage existing facilitators – a culture of data use, leadership, and accountability – to innovate and adapt in response to the new threat of COVID-19. This demonstrates the potential value of opportunities for innovation that come with an unprecedented health system shock [26]. For example, innovation and adaptation to unique barriers during COVID-19, including lockdown and movement restrictions, likely led both countries (and many other places) to more rapidly expand their use of telemedicine and digital health than likely would have happened otherwise. Adoption of digital health and telehealth was already under way, albeit more slowly, in many parts of the world before the COVID-19 pandemic [27], and accelerated because of it [28,29].

Across the two time periods, societal trust, reflected by high acceptability and a culture of accountability and leadership, seemed to correlate with higher trust during the COVID-19 period [30]. This higher trust could have had an impact in helping to reduce the disruptions to care-seeking experienced in Rwanda vs Bangladesh and likely contributed to increased resilience capabilities such as through integrated, diverse, and transformative capacities [7,31,32]. Conversely, countries with lower trust within society tended to be less resilient, as measured by higher caseloads and deaths during the COVID-19 pandemic [33].

Health insurance and efforts to achieve universal health coverage (UHC) appeared to be an important facilitator for Rwanda, but a barrier for Bangladesh. There, low insurance coverage and high out-of-pocket expenses were cited both as barriers to lowering under-five mortality and to maintaining access during COVID-19. During the first year of COVID-19, countries with higher UHC coverage seemed to do better in limiting disruptions and ensuring ongoing access to essential services [34,35], while some countries with low health insurance coverage saw increases in catastrophic health expenditures [36]. In some countries, COVID-19 responses included increased UHC-like provisions [35]; the pandemic highlighted the value of UHC in helping maintain or even expand access to essential health services [9,37,38].

Using mixed methods implementation research allowed us to identify strategies and transferable lessons that can be adopted and adapted by other countries, even those with more resilient health systems learning and capacity development, where it could contribute to resiliency in future shocks. The University of Global Health Equity in Rwanda is helping to provide that bridge by connecting implementers and policymakers in francophone African countries that have struggled in both the MDG and COVID-19 periods with the implementation research skills to identify and address gaps in health system resilience through strengthening existing or introducing new strategies which reflect their context, and which are adapted for use in their countries, focussing on the delivery of EBIs known to reduce amenable under-five mortality.

Our study had several limitations. For example, an important limitation to the scope of our research was that we were unable to conduct a rigorous systematic review and, limited by time and resources, only studied two countries. Although the cross-case analysis methodology helped to strengthen the evidence for the findings of common implementation strategies and facilitators, without more comparison countries – including those less successful in reducing under-five mortality during the MDG period or in maintaining essential health services during the COVID-19 period – we must interpret these conclusions with care and encourage work in other countries replicating these case study methods to further validate the findings. In addition, while these were positive examples, we did not also study countries which did not exhibit resiliency to identify shared strategies. Like all qualitative research, our data were reported by our KIs, who relied on recall, which means that bias cannot be excluded.

## CONCLUSIONS

The strategies and contextual factors Bangladesh and Rwanda leveraged to build ‘everyday resilience’ before COVID-19 likely supported the maintained delivery of the EBIs targeting under-five mortality during the early stages of the pandemic. Strengthening contextual factors such as leadership and governance; a culture of accountability; and a strong pre-existing community health system and structure likely contributed to these countries’ resilience capabilities. Likewise, the use of implementation strategies such as data use for decision-making, the use of digital platforms and e-health, and building on CHW programmes and community-based health care delivery likely had the same effect. Expanding understanding of pre-existing factors and strategies that contributed to resilience before and during the pandemic is important to support other countries’ efforts to incorporate ‘everyday resilience’ into their health systems.
